# Integrated Transcriptomic and Metabolomic Analysis of *OsMRS2-6* in Rice Growth and Chloroplast Traits

**DOI:** 10.3390/genes17101191

**Published:** 2026-09-26

**Authors:** Yongli Zhu, Jinghong Zhang, Hongcai Li, Zaihui Zhou, Yongjun Lin, Fei Zhou

**Affiliations:** 1 National Key Laboratory of Crop Genetic Improvement, Huazhong Agricultural University, Wuhan 430070, China; zhuyongli@webmail.hzau.edu.cn (Y.Z.); lihongcai1234@126.com (H.L.); zaihuizhou@mail.hzau.edu.cn (Z.Z.); 2 Key Laboratory of Plant Genetics and Molecular Breeding, Zhoukou Normal University, Zhoukou 466001, China; jinghongzh@outlook.com; 3 Hubei Hongshan Laboratory, Huazhong Agricultural University, Wuhan 430070, China

**Keywords:** *OsMRS2-6*, chloroplast function, MRS2/MGT family, transcriptomics, metabolomics

## Abstract

**Background/Objectives:** OsMRS2-6 is a member of the MRS2/MGT family, but its relationship with chloroplast status and growth and development in rice remains incompletely understood. **Methods:** In this study, we generated *OsMRS2-6* knockout and overexpression lines and combined transient subcellular localization, chloroplast ultrastructural analysis, plastid gene-expression profiling, transcriptomics and metabolomics to investigate the biological functions of *OsMRS2-6*. **Results:** OsMRS2-6 was localized to chloroplasts and was preferentially expressed in mature flag leaves. Loss of *OsMRS2-6* was associated with reduced seedling growth, plant dwarfing, disorganized thylakoid structures and increased plastoglobule abundance. In addition, *OsMRS2-6* knockout altered the editing efficiency of eight chloroplast RNA-editing sites and disrupted the expression of genes associated with NADH dehydrogenase-like complexes, photosystems and chloroplast ribosomes. By contrast, *OsMRS2-6* overexpression was associated with earlier heading and increased bleaching of panicle tissues. Transcriptomic and metabolomic analyses identified 691 shared differentially expressed genes and 270 shared differential metabolites in the knockout-versus-wild-type and overexpression-versus-wild-type comparisons, respectively. Integrated analysis further identified 11 pathways jointly involving genes and metabolites, mainly related to sugar metabolism, energy production, amino-acid turnover, antioxidant metabolism and secondary metabolism. **Conclusions:** Together, these findings extend previous knowledge of OsMRS2-6/OsMGT3 beyond its reported roles in chloroplast Mg^2+^ uptake and photosynthetic regulation by associating altered *OsMRS2-6* expression with chloroplast ultrastructure, plastid RNA editing, reproductive-stage phenotypes and coordinated transcriptomic and metabolomic responses in rice.

## 1. Introduction

Chloroplasts are the primary sites of light-energy conversion in plants and also support the biosynthesis of hormones and metabolites required for plant growth and development [1]. Chloroplast biogenesis and functional maintenance depend on coordinated gene expression between the nuclear and plastid genomes, as well as the proper assembly and homeostasis of the chloroplast membrane system [2]. Magnesium ions (Mg^2+^) are indispensable mineral nutrients for these processes. Within chloroplasts, Mg^2+^ serves both as the central ion of chlorophyll and as a cofactor or activator for numerous enzymes involved in carbon fixation and other metabolic reactions [3]. Because the chloroplast envelope separates the stroma from the cytosol, the establishment and dynamic regulation of stromal Mg^2+^ concentrations require dedicated membrane transport systems. The MRS2/MGT family, which is homologous to CorA proteins, constitutes an important component of the plant Mg^2+^ transport system. Plant MRS2 homologues were first identified through their ability to complement functional defects in yeast mitochondrial mutants [4]. Subsequent studies of the *Arabidopsis* MRS2/MGT family showed that its members participate in Mg^2+^ uptake and intracellular distribution [5], with some also mediating Mg^2+^ transport between the cytosol and organelles [6]. In *Arabidopsis*, AtMRS2-11/MGT10 was identified as a Mg^2+^ transporter localized to the chloroplast inner envelope membrane [7]. Loss of MGT10 function causes defective chloroplast development, disorganized thylakoid structures and impaired photosynthetic performance [8,9]. In addition, the inner-envelope proteins MGR8 and MGR9 contribute to chloroplast Mg^2+^ transport, and their simultaneous loss severely affects thylakoid formation and photosynthetic-complex assembly [10]. These studies indicate that precise regulation of chloroplast Mg^2+^ transport is fundamental to the maintenance of chloroplast structure and functional homeostasis. The functions of individual chloroplast Mg^2+^ transporters in Mg^2+^ homeostasis and photosynthetic regulation are not identical, suggesting functional diversification within this family [3].

Nine OsMRS2 family genes have been identified in rice (*Oryza sativa*) and classified into five phylogenetic clades. *OsMRS2-6*, also known as *OsMGT3*, corresponds to the locus *Os03g0684400* and encodes a chloroplast-localized Mg^2+^ transporter. *OsMRS2-6* expression increases during leaf maturation and exhibits diurnal variation in fully expanded leaves [11]. Genetic studies have shown that OsMGT3 contributes to chloroplast Mg^2+^ uptake and its diurnal fluctuation, and affects Rubisco activity, photosynthetic carbon assimilation and vegetative growth [12]. Its rhythmic expression is negatively regulated by the circadian-clock components OsPRR59 and OsPRR95, thereby linking chloroplast Mg^2+^ homeostasis with carbon fixation and sugar export [13]. Recent work has further shown that loss of *OsMGT3* increases cytosolic Mg^2+^ levels, induces the expression of phenylalanine ammonia-lyase genes and salicylic-acid accumulation, and enhances rice resistance to the blast fungus *Magnaporthe oryzae* and the bacterial blight pathogen *Xanthomonas oryzae* pv. *oryzae* [14].

Previous studies have established that *OsMRS2-6*, also known as *OsMGT3*, contributes to chloroplast Mg^2+^ uptake, Rubisco activity, photosynthetic carbon assimilation, vegetative growth, circadian regulation and disease resistance [12,13,14]. However, whether altered *OsMRS2-6* expression is associated with chloroplast ultrastructure, plastid RNA editing, reproductive-stage phenotypes and coordinated transcriptomic and metabolomic responses remains unclear. In this study, we generated CRISPR/Cas9-mediated *OsMRS2-6* loss-of-function mutants and overexpression lines, and found that altered *OsMRS2-6* expression was associated with changes in chloroplast gene expression, chloroplast RNA-editing efficiency, chloroplast ultrastructure and plastoglobule accumulation, together with abnormalities in vegetative and reproductive development, including earlier heading and bleaching of panicle tissues in the overexpression materials. Integrated transcriptomic and metabolomic analyses further identified candidate gene-expression and metabolic changes accompanying these phenotypes. Thus, the present study extends the functional context of *OsMRS2-6* beyond its established roles in chloroplast Mg^2+^ transport and photosynthetic regulation by linking altered *OsMRS2-6* expression with chloroplast structural changes, plastid RNA editing, reproductive-stage phenotypes and coordinated molecular responses.

## 2. Materials and Methods

### 2.1. Plant Materials and Growth Conditions

Rice cultivar Zhonghua11 (*O sativa* L. ssp. *japonica* cv. Zhonghua11, ZH11) was used as the wild-type (WT) genetic background. The knockout mutants and overexpression lines were generated in the ZH11 background. Field experiments were conducted at the experimental farm of Huazhong Agricultural University (30.4° N, 114.2° E), where all materials were grown under the same field-management conditions. T_1_-generation materials used for loss-of-function phenotyping were grown under tissue-culture conditions. After surface sterilization, the seeds were inoculated onto half-strength Murashige and Skoog (MS) medium and germinated and grown at 28 °C under a 14 h light/10 h dark photoperiod. For phenotypic analyses, T_2_-generation seeds of the *OsMRS2-6* overexpression and knockout lines were sown simultaneously with WT seeds, and the seedlings were transplanted to the experimental field at the three-leaf stage. Three independent families were selected for each knockout and overexpression material, with 30 plants grown per family and arranged in rows of 10 plants.

### 2.2. Generation of OsMRS2-6 Knockout and Overexpression Materials

The *osmrs2-6* knockout materials were generated using the CRISPR/Cas9 system. One pair of single-guide RNAs (sgRNAs) targeting *OsMRS2-6* was designed using the CRISPR-P v2.0 online tool and cloned into the pYLCRISPR/Cas9-MH vector. The recombinant vectors were introduced into ZH11 calli by Agrobacterium-mediated transformation to generate T_0_ transgenic plants. T_0_ plants were screened by PCR amplification and sequencing, and homozygous mutants carrying frameshift mutations predicted to generate premature termination codons were selected for subsequent analyses. Primer sequences are listed in Supplementary Table S1.

The OE-OsMRS2-6 overexpression materials were generated by cloning the full-length *OsMRS2-6* coding sequence (CDS) into an expression vector. The recombinant vector was introduced into ZH11 calli by Agrobacterium-mediated transformation [15]. The resulting transgenic plants were verified by PCR and used for expression analysis and phenotypic characterization. Primer sequences are listed in Supplementary Table S1.

### 2.3. Phylogenetic Tree Construction and Multiple-Sequence Alignment

To investigate the evolutionary relationships among OsMRS2-6 homologues, homologous protein sequences were retrieved from the NCBI database. Based on the corresponding homology annotations and taxonomic coverage, representative sequences were selected for phylogenetic analysis to ensure broad representation of the sampled taxa. Multiple-sequence alignment was performed using MAFFT v7.490 with the --localpair --maxiterate 1000 options [16], and the resulting alignment was trimmed using trimAl v1.5.rev0 with the -strictplus option [17]. Substitution-model selection was performed using ModelFinder implemented in IQ-TREE v3.1.2, and the best-fitting model was determined to be Q.INSECT+F+I+G4 according to the Bayesian information criterion (BIC). A maximum-likelihood phylogenetic tree was constructed using IQ-TREE v3.1.2, with 1000 ultrafast bootstrap replicates and 1000 SH-aLRT replicates used to assess node support [18]. The resulting tree was annotated and graphically refined using the online iTOL tool. Sequence alignment inspection and visualization were performed using Jalview 2.11.4.0 [19]. To assess sequence conservation among rice MRS2 family proteins, rice MRS2 family protein sequences were retrieved from the NCBI database and subjected to multiple-sequence alignment and comparative analysis. Protein accession numbers are provided in Supplementary Tables S2 and S3.

### 2.4. Subcellular Localization

The full-length *OsMRS2-6* coding sequence (CDS), excluding the stop codon, was cloned into the pAN580 expression vector to generate a 35S-OsMRS2-6-GFP fusion construct. The recombinant construct was introduced into rice protoplasts, which were incubated in the dark for 12–16 h. Fluorescence signals were subsequently detected using a Zeiss LSM980 super-resolution microscope equipped with Airyscan2 (ZEISS Microscopy, Oberkochen, Germany).

### 2.5. Transmission Electron Microscopy Analysis of Chloroplast Ultrastructure

Leaves from WT, *osmrs2-6* and OE-OsMRS2-6 plants at the three-leaf stage were cut into small pieces and immediately fixed in 2.5% glutaraldehyde. The samples were thoroughly infiltrated under vacuum, washed with phosphate-buffered saline (PBS) and post-fixed with 1% osmium tetroxide. The samples were then dehydrated through a graded ethanol series, infiltrated with resin and embedded. Ultrathin sections were prepared and stained before chloroplast ultrastructure was examined using a transmission electron microscope operated at 120 kV at the Electron Microscopy Facility (Hitachi High-Tech Corporation, Tokyo, Japan) of Huazhong Agricultural University.

### 2.6. Multiplex RT–PCR–Mass Spectrometry Analysis of Chloroplast RNA-Editing Efficiency

Leaves from WT, *osmrs2-6-1*, *osmrs2-6-2* and *osmrs2-6-3* plants at the three-leaf stage were collected for total RNA extraction and reverse transcription to generate cDNA. Based on previously reported organellar RNA-editing sites in rice, 20 chloroplast RNA-editing sites and 8 mitochondrial RNA-editing sites were examined. Multiplex PCR primers and single-base extension primers were designed according to approximately 200 bp sequences flanking each site.

Following treatment with shrimp alkaline phosphatase (SAP) and exonuclease I, the PCR products were subjected to an iPLEX single-base extension reaction. The extension products were purified, transferred onto MassARRAY SpectroCHIP arrays and analyzed using a MassARRAY 1.40.0 mass spectrometry system. Mass-spectrometry data were analyzed with TYPER 4.0 software to obtain the peak areas of edited products, area (*T*), and unedited products, area (*C*). RNA-editing efficiency was calculated using the following equation:

RNA-editing efficiency (%) = area (*T*)/[area (*T*) + area (*C*)] × 100%

where area (*T*) represents the peak area of the edited product and area (*C*) represents the peak area of the unedited product.

### 2.7. qRT-PCR

Total RNA was extracted from leaves of WT, *osmrs2-6-1*, *osmrs2-6-2* and *osmrs2-6-3* plants at the three-leaf stage using TRIzol reagent (TransGen Biotech). After DNase treatment, cDNA was synthesized using M-MLV reverse transcriptase (Invitrogen) and random primers. Quantitative real-time PCR was performed using a ViiA™ 7 Real-Time PCR System with a 384-Well Block. The reaction program consisted of an initial denaturation step at 95 °C for 10 min, followed by 40 cycles of 95 °C for 15 s and 60 °C for 1 min. *Actin* was used as the reference gene, and relative transcript abundance was calculated using the 2^−ΔΔCt^ method [20]. The experiment was performed in three replicates. Primer sequences used for qRT–PCR are listed in Supplementary Tables S1 and S4.

### 2.8. Transcriptome Sequencing and Analysis

Transcriptome analysis was performed using leaves collected from T_2_-generation WT, *osmrs2-6* and OE-OsMRS2-6 plants at the three-leaf stage. Three independent biological replicates were included for each group, resulting in nine sequencing samples in total. Each biological replicate consisted of a pooled leaf sample from the corresponding material. Total RNA was extracted using TRIzol, and its quality and integrity were assessed using a NanoDrop 2000 (Thermo Fisher Scientific, Waltham, MA, USA) spectrophotometer and an Agilent 2100 Bioanalyzer (Agilent Technologies, Santa Clara, CA, USA). Samples with an RIN of ≥7.0 and a 28S/18S ratio of ≥1.0 were used for mRNA library construction. Messenger RNA was enriched using Oligo (dT) magnetic beads, and paired-end sequencing was performed on an Illumina platform with a read length of 150 bp (PE150).

Raw reads were quality-filtered using fastp v0.23.2 by removing reads containing adapter sequences, reads with more than 10% ambiguous bases (N), and reads in which more than 50% of the bases had a quality score of Q ≤ 10. The filtered clean reads were aligned to the rice reference genome (*O sativa* MSU v7.0) using HISAT2 v2.2.1, followed by transcript assembly and gene-level read counting using StringTie v2.2.1 with the corresponding MSU v7.0 gene-structure annotation. FPKM values were used only for gene-expression presentation and sample-correlation analysis. Differential-expression analysis was performed using the unnormalized gene-level raw integer Count matrix generated through the StringTie-based workflow, rather than FPKM values, with DESeq2 v1.42.0. Differentially expressed genes were identified using the criteria of an absolute log2 fold change of ≥1 and an adjusted *p* value (FDR) < 0.05. GO and KEGG enrichment analyses were performed using the clusterProfiler R 4.1.1 package, with *p* < 0.05 considered statistically significant.

### 2.9. Metabolomic Sequencing and Analysis

Untargeted metabolomic profiling of rice leaves was performed using a UPLC-QTOF-MS platform. Leaves from three-leaf-stage WT, *osmrs2-6* and OE-OsMRS2-6 plants were analyzed, with three independent biological replicates for each group. Data were acquired using a Waters Acquity I-Class PLUS (Waters Corporation, Milford, MA, USA) ultra-performance liquid chromatography system coupled to a Waters Xevo G2-XS QTOF (Waters Corporation, Milford, MA, USA) mass spectrometer. Raw data were acquired using MassLynx v4.2 and processed using Progenesis QI v2.4 for peak extraction, peak alignment and normalization. Pooled QC and reagent-blank samples were included, and samples were injected in randomized order. Data preprocessing included feature extraction, alignment, filtering, missing-value processing, normalization and QC-based correction. Relative metabolite abundance was represented by chromatographic peak area. Global metabolic differences among samples were evaluated using principal-component analysis (PCA). OPLS-DA models were constructed using the ropls R package and evaluated by 7-fold cross-validation and 200 permutation tests. Differential metabolites were identified using two-sided independent-samples *t*-tests with Benjamini–Hochberg correction. Metabolites with an absolute log2 fold change ≥ 1, adjusted FDR < 0.05 and VIP ≥ 1 were considered differential metabolites.

### 2.10. Integration of Transcriptomic and Metabolomic Data

For the integrated transcriptomic and metabolomic analysis, fold changes were calculated as the abundance in OE-OsMRS2-6 divided by that in WT for the OE-OsMRS2-6 versus WT comparison, and as the abundance in *osmrs2-6* divided by that in WT for the *osmrs2-6* versus WT comparison. Upregulated genes in the OE-OsMRS2-6 versus WT comparison and downregulated genes in the *osmrs2-6* versus WT comparison were selected using an FC threshold of 1.5 and an FDR threshold of 0.01. The intersection of these two gene sets was defined as the target gene set.

For metabolites, differential features were selected using the criteria of an absolute log2 fold change of ≥1, an adjusted FDR < 0.05 and an OPLS-DA VIP value of ≥1. The intersection of metabolites with higher abundance in OE-OsMRS2-6 than in WT and lower abundance in *osmrs2-6* than in WT was defined as the target metabolite set. The corresponding gene-expression and metabolite-abundance data were integrated into a joint feature matrix and subjected to principal component analysis (PCA) in R. The PCA results were visualized using the ggplot2 package.

### 2.11. Statistical Analysis

The materials used in this study comprised three groups: WT, *osmrs2-6* knockout and OE-OsMRS2-6 overexpression materials. The corresponding line identifiers were WT, *osmrs2-6-1*, *osmrs2-6-2*, *osmrs2-6-3*, OE-OsMRS2-6-1, OE-OsMRS2-6-2 and OE-OsMRS2-6-3. These materials were used for RNA-editing efficiency analysis, field phenotype evaluation, transmission electron microscopy, transcriptome sequencing and metabolomic analysis.

All experiments included at least three biological replicates. Data are presented as the mean ± standard deviation. Comparisons between two groups were performed using Student’s *t*-test, whereas comparisons among multiple groups were performed using one-way analysis of variance (ANOVA) followed by a post hoc multiple-comparisons test. *p* < 0.05 was considered statistically significant, and *p* < 0.01 was considered highly statistically significant.

## 3. Results

### 3.1. OsMRS2-6 Is Evolutionarily Conserved and Contributes to Normal Seedling Growth in Rice

To investigate the biological function of *OsMRS2-6*, we generated two independent homozygous knockout lines, *osmrs2-6-1* and *osmrs2-6-2*, with consistent genotypes using a CRISPR/Cas9 system targeting two sites in *OsMRS2-6* (Figure 1a). Sequence analysis showed that both mutants carried frameshift mutations predicted to generate premature termination codons. We also obtained an additional knockout material carrying frameshift mutations in both alleles but displaying genotype heterogeneity. Under normal growth conditions, all knockout materials exhibited reduced seedling height, restricted leaf expansion and a consistent dwarf phenotype compared with the wild type (WT) (Figure 1b). These results indicate that loss of *OsMRS2-6* function is associated with reduced vegetative growth during the rice seedling stage.

We next analyzed the tissue-expression pattern of *OsMRS2-6* by qRT–PCR. *OsMRS2-6* transcript abundance was highest in mature flag leaves, followed by that in young leaves, whereas relatively lower expression levels were detected in panicles, roots and stems (Figure 1c). Compared with flag leaves, *OsMRS2-6* expression was significantly lower in the other four tissues (*p* < 0.01). The preferential expression of *OsMRS2-6* in mature flag leaves suggests that OsMRS2-6 may function predominantly in mature photosynthetic tissues.

Phylogenetic analysis showed that MRS2 homologues generally clustered according to taxonomic group. Rice OsMRS2-6 clustered closely with homologues from monocot species, including maize and wheat (Figure 1d). Multiple-sequence alignment of the nine rice MRS2 family members revealed a high degree of conservation in the C-terminal region, including the conserved GMN motif and the core region associated with Mg^2+^ transport, whereas the N-terminal region was comparatively more divergent (Figure 1e). These results indicate that rice MRS2 family proteins share conserved molecular features associated with Mg^2+^ transport, whereas their variable regions may contribute to functional diversification among family members.

### 3.2. OsMRS2-6 Localizes to Chloroplasts, and Its Loss of Function Is Associated with Changes in Chloroplast Ultrastructure and RNA Editing

To determine the subcellular localization of OsMRS2-6, the 35S-OsMRS2-6-GFP fusion construct and the 35S-GFP control construct were transiently introduced into rice protoplasts. GFP expressed alone was broadly distributed throughout the cytoplasm and showed little overlap with chlorophyll autofluorescence. By contrast, the green fluorescence of OsMRS2-6-GFP strongly overlapped with chlorophyll autofluorescence and was predominantly enriched at the chloroplast periphery (Figure 2a), indicating that OsMRS2-6 localizes to chloroplasts.

Transmission electron microscopy further revealed clear differences in chloroplast ultrastructure among WT, *osmrs2-6* and OE-OsMRS2-6 plants. Chloroplasts in WT plants contained regularly arranged thylakoid lamellae and few plastoglobules, whereas chloroplasts in *osmrs2-6* plants exhibited loosely arranged and locally disorganized thylakoid membranes, together with substantial plastoglobule accumulation. The thylakoid structures of OE-OsMRS2-6 chloroplasts were generally intact, although some chloroplasts also showed an increase in plastoglobule abundance (Figure 2b). Across three replicate groups, the mean number of plastoglobules per chloroplast was 2.43, 3.57 and 2.83 in WT; 6.86, 7.00 and 7.50 in *osmrs2-6*; and 3.43, 2.43 and 5.17 in OE-OsMRS2-6, respectively (Table 1). Thus, the number of plastoglobules per chloroplast was consistently higher in *osmrs2-6* mutants than in WT across all three replicate groups, whereas OE-OsMRS2-6 plants showed relatively modest but more variable plastoglobule accumulation.

We next used multiplex reverse-transcription PCR coupled with mass spectrometry (MRMS) to investigate the effects of *OsMRS2-6* loss of function on organellar RNA editing. Among the 20 chloroplast RNA-editing sites and eight mitochondrial RNA-editing sites examined, editing efficiency was altered at eight chloroplast sites in all three *osmrs2-6* mutant lines (Table 2). Compared with WT, editing efficiency at *ndhB*-196 increased from 75% to 96–100%, that at *ndhF*-21 increased from 70% to 82–100%, that at *rpoB*-182 increased from 51% to 99–100%, that at *rpoB*-156 increased from 83% to 100%, that at *rps8*-61 increased from 64% to 95–100%, that at *rps3*-170 increased from 52% to 76–82%, and that at *rps3*-955 increased from 68% to 80–95%. By contrast, editing efficiency at *ndhD*-293 decreased from 79% in WT to 57–65% in the mutant lines. These results show that loss of OsMRS2-6 function is associated with altered chloroplast RNA-editing efficiency, with seven of the eight affected sites showing increased editing efficiency.

### 3.3. Loss of OsMRS2-6 Function Alters the Expression of Chloroplast Genes Associated with Photosystems and Ribosomes

To determine whether the chloroplast structural abnormalities in *osmrs2-6* mutants were accompanied by changes in chloroplast gene expression, we used qRT–PCR to measure the relative transcript abundance of genes associated with the NADH dehydrogenase-like (NDH) complex, photosystems and chloroplast ribosomes. Among the genes encoding components of the NDH complex, the transcript levels of *ndhJ* and *ndhE* increased to approximately 1.6-fold and 1.1-fold of the WT levels, respectively, whereas those of *ndhD* and *ndhF* decreased to approximately 0.7-fold and 0.9-fold of the WT levels, respectively (Figure 3a).

Photosystem-related genes also showed changes in transcript abundance in different directions. The transcript levels of *psaC* and *psbL* increased to approximately 1.2-fold and 1.8-fold of the WT levels, respectively, whereas those of *psaA*, *psbH*, *psbK* and *psbN* decreased to approximately 0.6–0.8-fold of the WT levels (Figure 3b). Chloroplast ribosomal protein-coding genes also showed widespread changes. The transcript levels of *rpl32*, *rpl36* and *rps7* increased to approximately 1.3–1.4-fold of the WT levels, whereas those of *rpl22*, *rpl23*, *rpl33*, *rps12-1*, *rps14* and *rps8* decreased to approximately 0.5–0.7-fold of the WT levels. Notably, *rps16* transcript abundance in *osmrs2-6* was reduced to near-undetectable levels (Figure 3c). These changes in transcript abundance indicate that loss of *OsMRS2-6* function is accompanied by altered expression of chloroplast genes associated with photosynthetic electron transport, photosystem components and chloroplast translation.

### 3.4. OsMRS2-6 Overexpression Is Associated with Earlier Heading and Increased Bleaching of Panicle Tissues

To examine the association between altered *OsMRS2-6* expression and rice growth and development, WT plants, *OsMRS2-6* overexpression lines and *osmrs2-6* knockout mutants were grown simultaneously, and plant phenotypes were evaluated at maturity (Figure 4). WT plants exhibited a relatively uniform architecture, with comparable plant height, tiller number and heading time. Compared with WT plants, the *OsMRS2-6* overexpression lines showed no apparent changes in plant height or tiller number, but headed earlier and displayed pronounced bleaching in some panicle tissues. By contrast, the *osmrs2-6* mutants were characterized mainly by reduced plant height, weaker growth and delayed maturation. Quantification further showed that the number of bleached panicle tissues per plant ranged from 0 to 3 in WT plants and from 3 to 8 in the overexpression lines, with a maximum of eight tissues per plant (Table 3). These results indicate that *OsMRS2-6* overexpression is associated with earlier heading and increased bleaching of panicle tissues in rice.

### 3.5. Transcriptome Data Analysis

To characterize the transcriptional responses associated with altered *OsMRS2-6* expression, we performed transcriptome sequencing of leaves from WT, *osmrs2-6* knockout and OE-OsMRS2-6 overexpression materials. Principal-component analysis (PCA) showed that samples from the three genotypes occupied distinct regions in the two-dimensional coordinate space. PC1 and PC2 explained 33.9% and 20.23% of the transcriptomic variation, respectively, accounting for 54.13% of the total variance when combined (Figure 5a). The clear separation of WT, *osmrs2-6* and OE-OsMRS2-6 samples indicated that both loss and overexpression of *OsMRS2-6* were accompanied by extensive changes in the rice leaf transcriptome.

Differential-expression analysis identified 3105 differentially expressed genes (DEGs) in the *osmrs2-6* versus WT comparison and 1246 DEGs in the OE-OsMRS2-6 versus WT comparison. Of these, 691 DEGs were shared between the two comparisons (Figure 5b). Thus, loss of OsMRS2-6 was associated with a broader genotype-specific transcriptional response than overexpression, whereas both perturbations were also associated with a common set of responsive genes.

Gene ontology (GO) enrichment analysis of the 691 shared DEGs showed that these genes were mainly involved in the regulation of defense responses, jasmonic-acid-mediated signaling, hydrogen-peroxide catabolism, responses to oxidative stress and wounding, carbohydrate metabolism, intracellular iron sequestration and nitrate assimilation. Additional enriched terms included RNA biosynthesis, RNA metabolism, transcriptional regulation and negative regulation of cellular biosynthetic processes (Figure 5c). Among the Kyoto Encyclopedia of Genes and Genomes (KEGG) pathways shown in the figure, phenylpropanoid biosynthesis, plant-pathogen interaction, starch and sucrose metabolism, cyanoamino-acid metabolism, plant hormone signal transduction, flavonoid biosynthesis and carotenoid biosynthesis displayed relatively low *q* values (Figure 5d). Together, these results indicate that altered *OsMRS2-6* expression is associated with both shared and genotype-specific transcriptional responses, particularly those related to defense and hormone signaling, oxidative stress, and primary and secondary metabolism.

### 3.6. Metabolomic Data Analysis

Metabolomic analysis showed clear separation of the metabolic profiles of WT, *osmrs2-6* knockout and OE-OsMRS2-6 overexpression materials. PC1 and PC2 explained 32.5% and 31.6% of the total variance, respectively, accounting for 64.1% of the overall metabolomic variation (Figure 6a). Differential-metabolite analysis identified 795 differential metabolites in the *osmrs2-6* versus WT comparison and 691 differential metabolites in the OE-OsMRS2-6 versus WT comparison. Of these, 270 metabolites were shared between the two comparisons, whereas 525 and 421 metabolites were specific to the knockout and overexpression materials, respectively (Figure 6b). Chemical classification of the shared differential metabolites showed that prenol lipids were the most abundant category, followed by carboxylic acids and derivatives and organooxygen compounds. Other major categories included benzenoids, benzopyrans, coumarins and their derivatives, fatty acyls, and steroids and steroid derivatives (Figure 6c). KEGG analysis showed that the shared differential metabolites were associated with pathways involved in amino-sugar and nucleotide-sugar metabolism, oxidative phosphorylation, branched-chain amino-acid degradation, histidine and lysine metabolism, diterpenoid biosynthesis, glutathione metabolism, ascorbate and aldarate metabolism, and the biosynthesis of various plant secondary metabolites (Figure 6d). These results indicate that altered *OsMRS2-6* expression is associated with changes in multiple metabolic pathways, including sugar metabolism, amino-acid metabolism, antioxidant metabolism, and secondary metabolism. However, the transcriptomic and metabolomic data do not establish direct targets or direct regulatory relationships, and further experimental validation is required to determine the underlying mechanisms.

### 3.7. Integrated Transcriptomic and Metabolomic Analysis

Table S2 expression were related but not fully concordant (Figure 7a). Pathway-level comparison identified 11 pathways shared by the differentially expressed genes and differential metabolites, whereas 108 pathways were associated exclusively with the transcriptomic data and five pathways exclusively with the metabolomic data (Figure 7b). The shared pathways mainly included amino-sugar and nucleotide-sugar metabolism, glutathione metabolism, butanoate metabolism, amino-acid biosynthesis, ascorbate and aldarate metabolism, lysine degradation, branched-chain amino-acid degradation, tyrosine and tryptophan metabolism, diterpenoid biosynthesis and oxidative phosphorylation (Figure 7c). Amino-sugar and nucleotide-sugar metabolism showed a strong enrichment signal at the gene level and included a relatively large number of associated genes, whereas oxidative phosphorylation and branched-chain amino-acid degradation showed higher enrichment factors at the metabolite level. These results indicate that altered OsMRS2-6 expression is accompanied by coordinated changes at both the transcriptomic and metabolic levels, particularly in pathways related to sugar metabolism, energy metabolism, amino-acid turnover and antioxidant metabolism.

## 4. Discussion

Previous studies have shown that OsMRS2-6/OsMGT3 contributes to chloroplast Mg^2+^ uptake and its diurnal fluctuation, and affects Rubisco activity and photosynthetic carbon assimilation [12]. Together with the findings of the present study, these observations are consistent with a broader role of OsMRS2-6 in chloroplast organization and molecular function. An appropriate ionic environment is important for thylakoid assembly, plastid gene expression and photosynthetic metabolism, but the present study did not directly assess photosynthetic performance. Thus, the developmental effects associated with altered OsMRS2-6 expression may reflect combined changes in multiple chloroplast processes rather than a confirmed impairment of photosynthesis. Accordingly, the new contribution of the present study is not the identification of OsMRS2-6/OsMGT3 as a chloroplast Mg^2+^ transporter, but the association of altered OsMRS2-6 expression with chloroplast ultrastructural changes, plastid RNA-editing variation, reproductive-stage phenotypes and coordinated transcriptomic and metabolomic responses under the same genetic framework.

Loss of the *Arabidopsis* chloroplast Mg^2+^ transporters MGT10, MGR8 and MGR9 affects thylakoid biogenesis and photosynthetic-complex assembly [8,9,10], suggesting a conserved relationship between chloroplast Mg^2+^ homeostasis and maintenance of the membrane system. The chloroplast structural changes observed in the present study are consistent with this possibility, but they do not establish Mg^2+^ imbalance as the direct cause. First, neither bulk Mg^2+^ content in leaves nor Mg^2+^ concentration and subcellular distribution within chloroplasts were measured in this study. Therefore, the present data cannot determine whether altered *OsMRS2-6* expression changes chloroplast Mg^2+^ availability or whether such a change contributes directly to the observed phenotypes. Second, thylakoid remodeling and plastoglobule accumulation may also result from oxidative stress, altered lipid metabolism or photosynthetic damage. Plastoglobules participate in thylakoid-lipid remodeling and antioxidant regulation, and their accumulation may represent an adaptive response of chloroplasts to internal imbalance rather than an independent causal factor [21].

The relationship between OsMRS2-6 and plastid RNA metabolism requires careful definition. OsMRS2-6 is not a known RNA-editing factor, and the direction of change differed among the editing sites examined. Thus, the current evidence does not support a role for OsMRS2-6 in directly recognizing or catalyzing editing at specific RNA sites. The altered editing efficiencies may instead represent secondary consequences of altered chloroplast development or associated changes in the ionic, energetic or redox environment of chloroplasts. However, the present data do not distinguish among these possibilities, because they do not directly assess RNA-editing complex activity or a molecular interaction between OsMRS2-6 and the RNA-editing machinery. Plastid RNA processing, translation and photosynthetic-complex assembly are tightly coupled, and perturbation of one process may induce compensatory changes in the others. The RNA changes detected here should therefore be regarded as molecular signatures associated with OsMRS2-6 loss of function rather than evidence that OsMRS2-6 directly regulates plastid RNA metabolism.

Changes in chloroplast function may also be transmitted to whole-plant physiology through retrograde signaling. Chloroplast-derived metabolites, reactive oxygen species and redox signals can regulate nuclear-gene expression and thereby redistribute carbon, energy and antioxidant resources [22,23]. The multi-omics results obtained here are best interpreted as downstream or associated responses accompanying altered *OsMRS2-6* expression, rather than as evidence of direct regulation of a specific metabolic pathway. The coordinated changes observed in sugar and energy metabolism, amino-acid turnover, antioxidant processes and defense signaling may reflect an integrated response of rice to chloroplast perturbation. The finding that loss of *OsMGT3* affects cytosolic Mg^2+^ distribution, salicylic-acid accumulation and rice disease resistance [14] also indicates that this protein may influence processes beyond the chloroplast by altering cellular homeostasis. However, pathway enrichment can only suggest candidate relationships and cannot establish metabolic flux, regulatory direction or causality. Accordingly, the integrated omics results presented here identify the scope of physiological responses associated with altered *OsMRS2-6* expression, but do not identify direct molecular targets or establish that *OsMRS2-6* directly regulates sugar metabolism, amino-acid turnover, antioxidant metabolism or secondary metabolism.

Notably, enhancement of *OsMRS2-6* function does not necessarily produce phenotypes opposite to those caused by loss of function. Previous work showed that mesophyll-specific overexpression of *OsMGT3* can promote photosynthetic carbon assimilation and plant growth [12], whereas the overexpression materials examined here displayed a different reproductive-developmental response. This difference suggests that the effects of *OsMRS2-6* may depend jointly on expression dosage and tissue distribution. Moderate enhancement in mesophyll cells may benefit photosynthetic metabolism, whereas constitutive or sustained overexpression may affect chloroplast function, metabolite allocation, nutrient supply or developmental signaling in other tissues.

Bleaching of panicle tissues may be associated with altered *OsMRS2-6* expression, but it may also be influenced by developmental stage, cultivation conditions or biotic stress. Because Mg^2+^ content and chlorophyll content in panicle tissues were not measured, the underlying mechanism of this phenotype remains unresolved. In the absence of measurements of panicle chlorophyll and Mg^2+^ contents and pathogen assessments, this phenotype cannot be defined as a specific disease or a confirmed defect in chlorophyll biosynthesis.

## 5. Conclusions

In summary, the present study supports an association between *OsMRS2-6* expression and chloroplast function, cellular metabolism and rice growth and development. By integrating phenotypic observations, chloroplast ultrastructure, chloroplast RNA editing, plastid gene expression, transcriptomic data and metabolomic data from knockout and overexpression materials, this study expands the potential functional context of *OsMRS2-6* and establishes an association between altered *OsMRS2-6* expression, chloroplast status, metabolic reprogramming and developmental responses. The available evidence supports a working model in which altered *OsMRS2-6* expression is associated with changes in chloroplast structure, plastid gene expression, RNA editing and cellular metabolism, which may collectively accompany developmental responses. However, whether altered Mg^2+^ availability represents an initiating signal, and whether the observed molecular and phenotypic changes are direct effects or secondary responses, remain to be determined through measurements of Mg^2+^ content and subcellular distribution, photosynthetic-function measurements and tissue-specific expression analyses. In addition, the chloroplast ultrastructural, RNA-editing and multi-omics phenotypes reported here have not been specifically validated by complementation in the *osmrs2-6* mutant background.

## Figures and Tables

**Figure 1:**
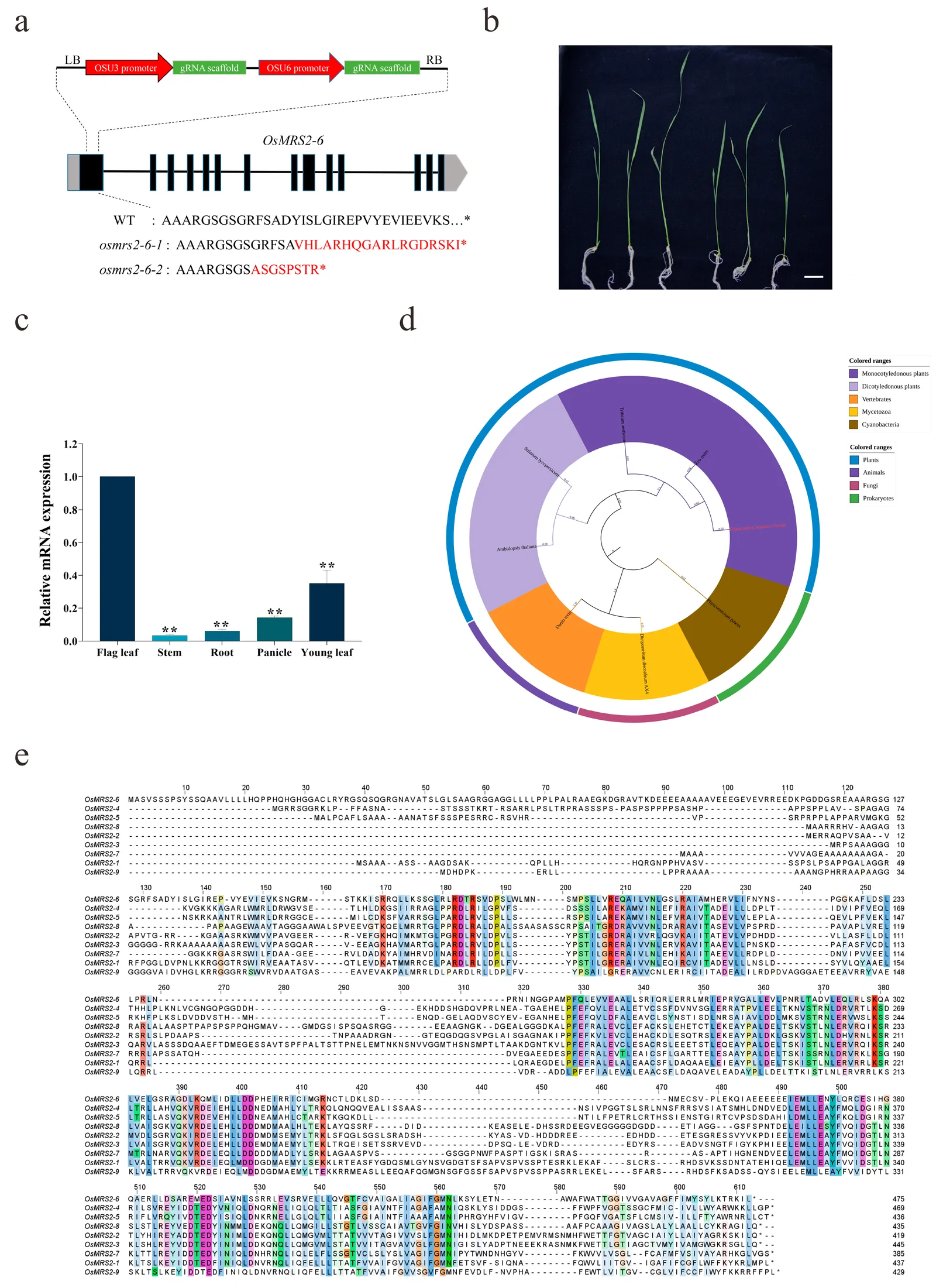
**Identification, tissue-expression pattern and evolutionary conservation of *OsMRS2-6* knockout mutants.** (**a**) Schematic representation of the *OsMRS2-6* gene structure and the CRISPR/Cas9 editing regions targeted at two sites. Black boxes indicate exons. The predicted protein sequences of WT, *osmrs2-6-1* and *osmrs2-6-2* are shown below. Red amino acids indicate aberrant sequences generated by frameshift mutations, and asterisks indicate premature termination codons. (**b**) Representative seedling phenotypes of WT and *osmrs2-6* mutants. Scale bar, 2 cm. (**c**) Relative *OsMRS2-6* transcript abundance in flag leaves, stems, roots, panicles and young leaves, determined by qRT–PCR. ** indicates *p* < 0.01. (**d**) Maximum-likelihood phylogenetic tree of MRS2 homologues from representative species. The colors of the outer ring indicate plants, animals, fungi and prokaryotes, whereas the inner sectors indicate monocots, dicots, vertebrates, slime molds and cyanobacteria. (**e**) Multiple-sequence alignment of the nine rice MRS2 family proteins. Background colors indicate the degree of amino-acid residue conservation.

**Figure 2:**
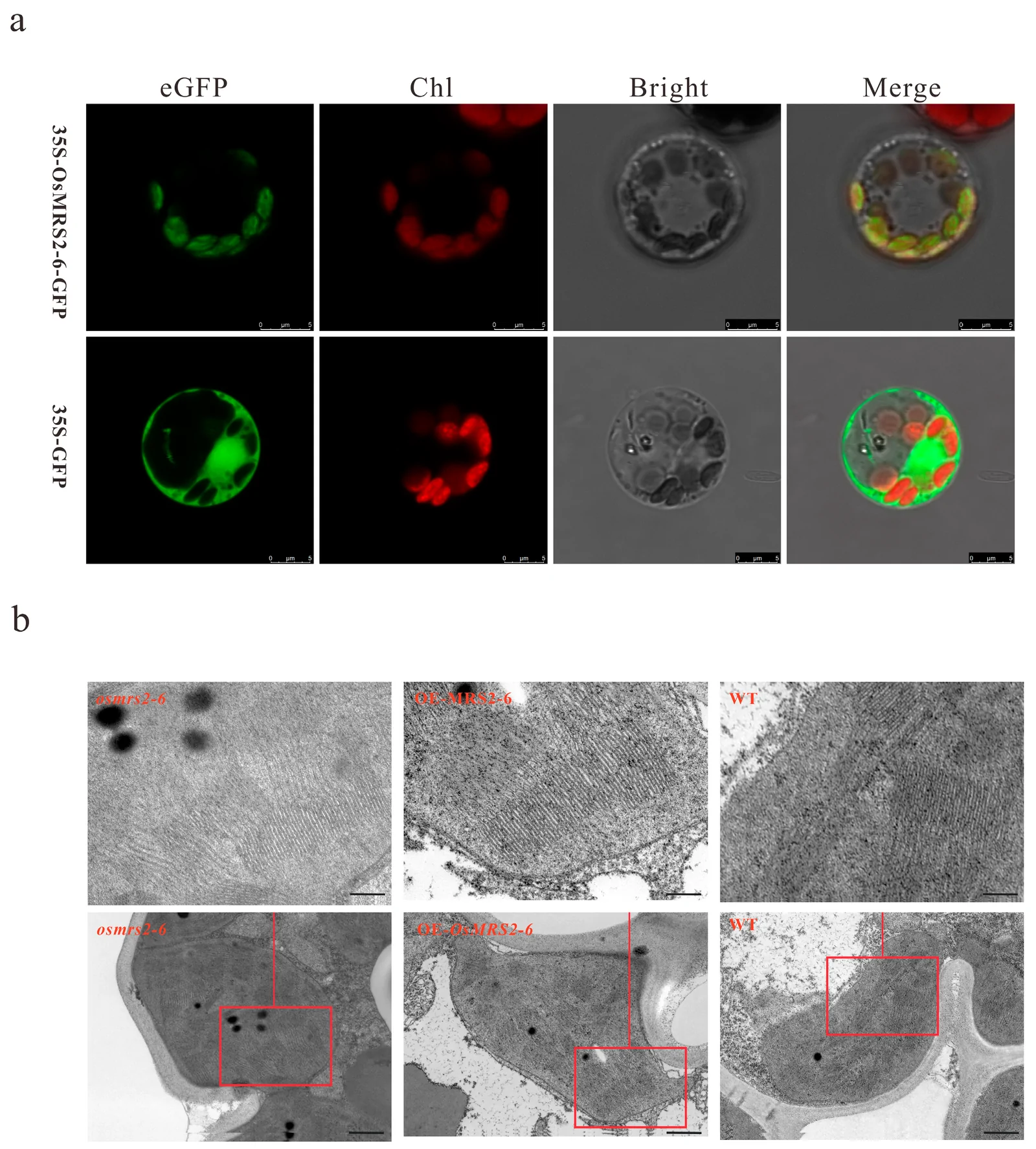
**Subcellular localization of OsMRS2-6 and chloroplast ultrastructure in different rice materials.** The red squares in the second row indicate the regions shown at higher magnification in the corresponding images in the first row. (**a**) Transient expression of the 35S-OsMRS2-6-GFP fusion protein and the 35S-GFP empty-vector control in rice protoplasts. GFP fluorescence, chlorophyll autofluorescence (Chl), bright-field images and merged images are shown from left to right. Scale bar, 5 μm. (**b**) Representative transmission electron microscopy images of chloroplasts in leaves from WT, *osmrs2-6* and OE-OsMRS2-6 plants. The boxed regions are shown at higher magnification in the adjacent images. Arrows indicate plastoglobules. Scale bars, 0.4 μm and 1 μm.

**Figure 3:**
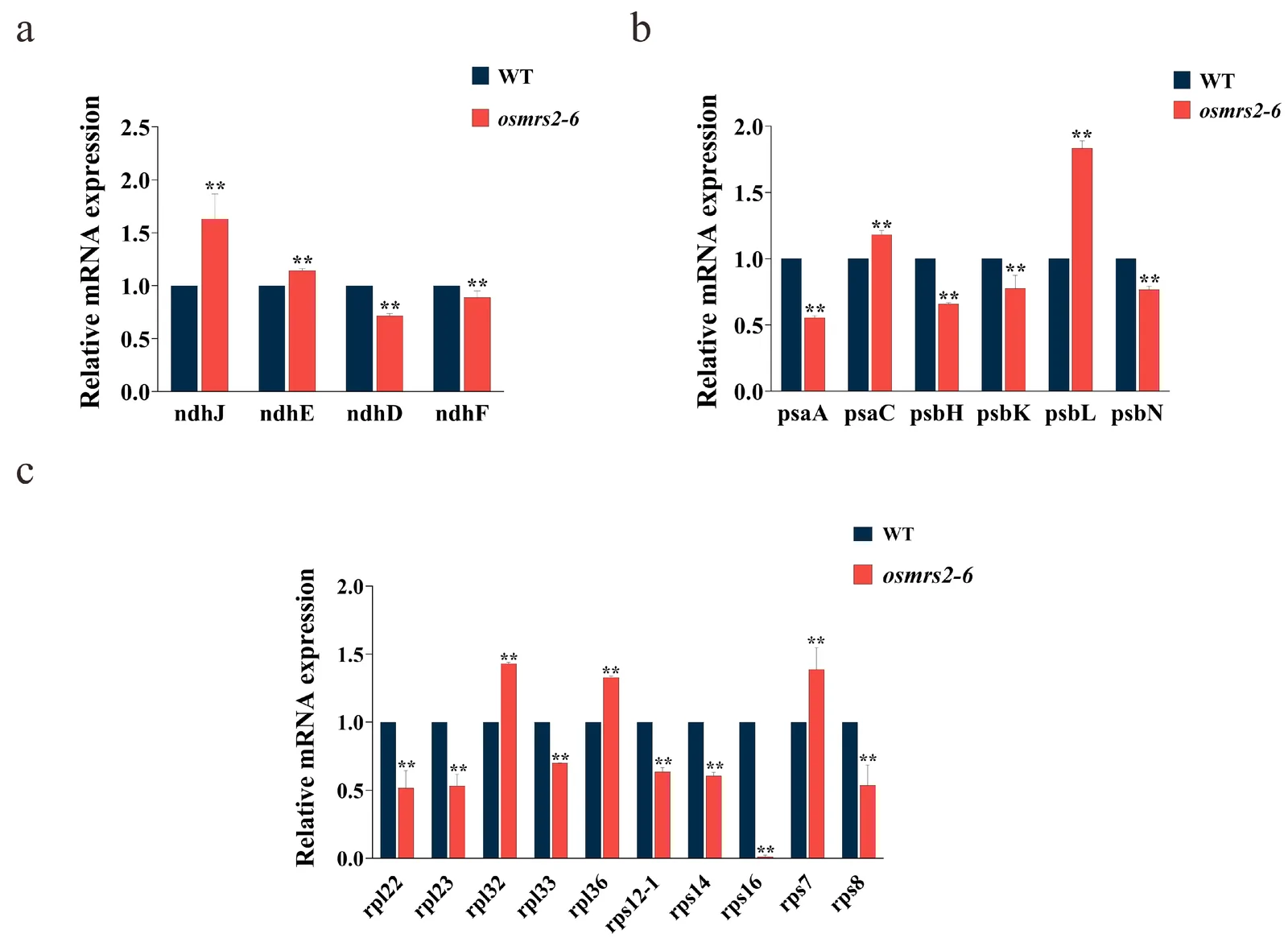
**Loss of *OsMRS2-6* function alters the expression of chloroplast genes associated with the NADH dehydrogenase-like complex, photosystems and ribosomes.** ** indicates *p* < 0.01. (**a**) Genes associated with the NADH dehydrogenase-like (NDH) complex, including *ndhJ*, *ndhE*, *ndhD* and *ndhF*. (**b**) Photosystem-related genes, including *psaA*, *psaC*, *psbH*, *psbK*, *psbL* and *psbN*. (**c**) Chloroplast ribosomal protein genes, including *rpl22*, *rpl23*, *rpl32*, *rpl33*, *rpl36*, *rps12-1*, *rps14*, *rps16*, *rps7* and *rps8*.

**Figure 4:**
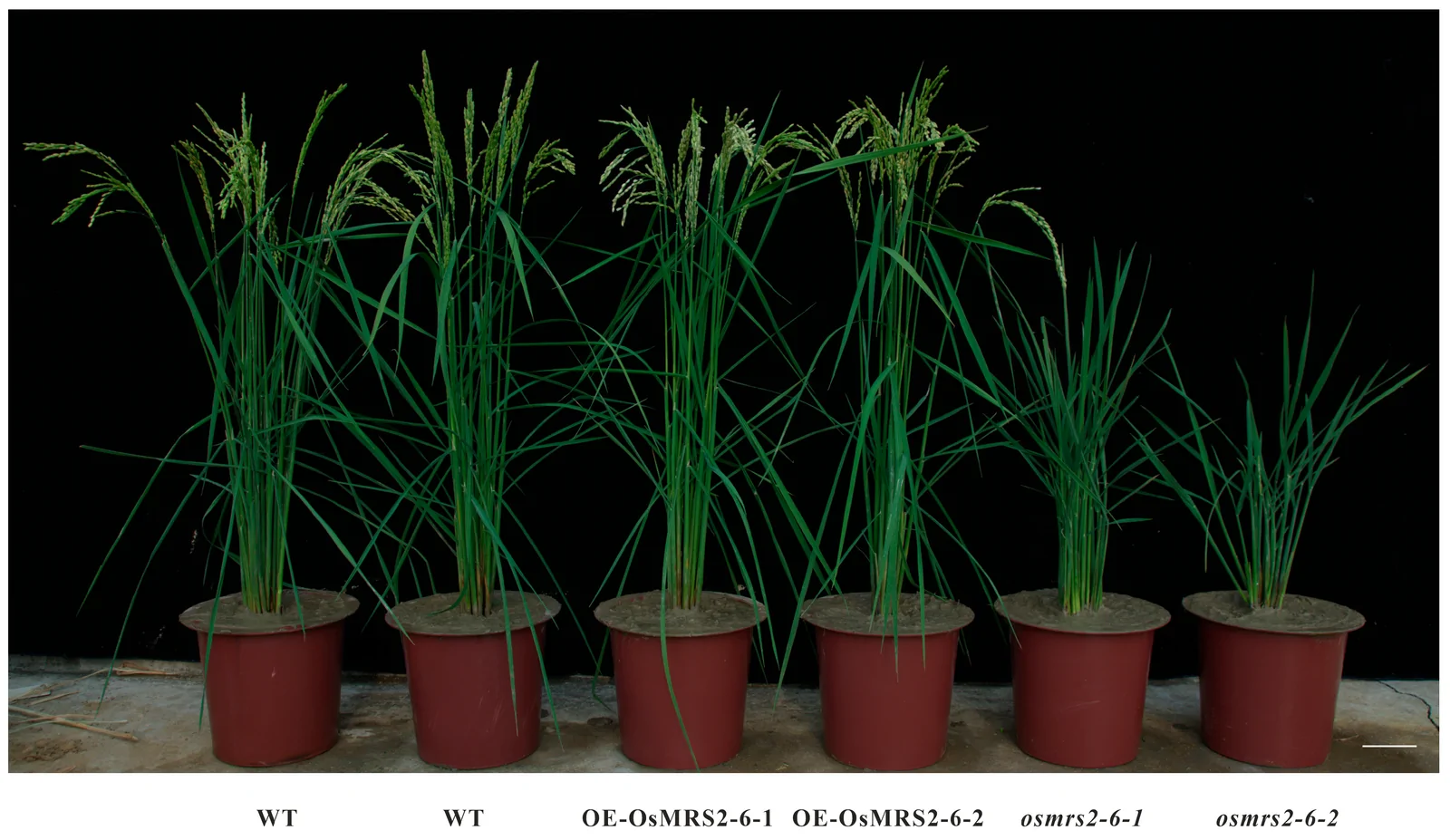
**Mature-stage phenotypes of WT, OE-OsMRS2-6 and *osmrs2-6* rice plants.** Scale bar, 10 cm.

**Figure 5:**
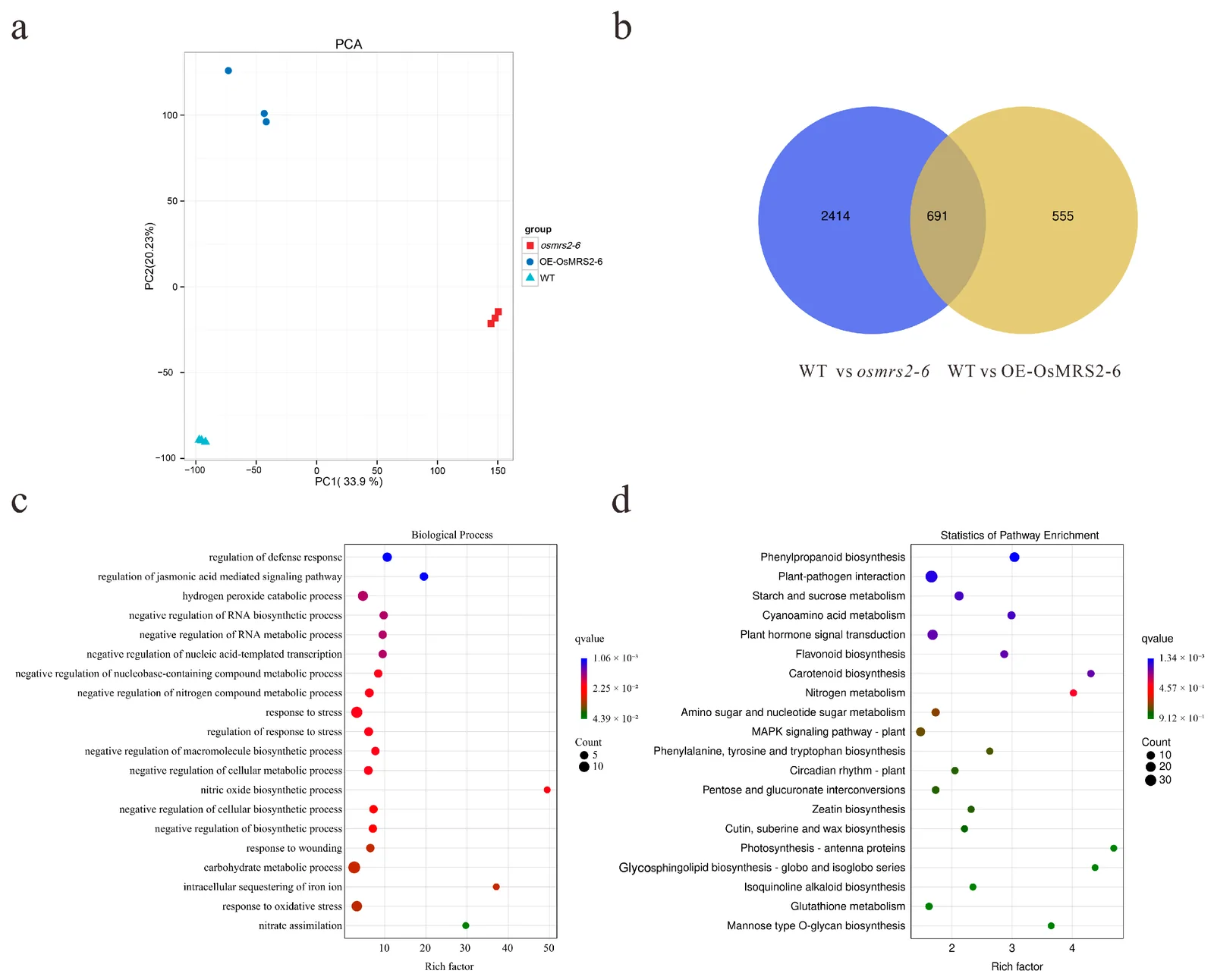
**Transcriptome analysis of leaves from WT, *osmrs2-6* and OE-OsMRS2-6 plants.** (**a**) Principal-component analysis (PCA) based on the global gene-expression profiles. (**b**) Venn diagram showing the differentially expressed genes (DEGs) identified in the *osmrs2-6* versus WT and OE-OsMRS2-6 versus WT comparisons. (**c**) Gene ontology (GO) biological-process enrichment analysis. (**d**) Kyoto Encyclopedia of Genes and Genomes (KEGG) pathway enrichment analysis.

**Figure 6:**
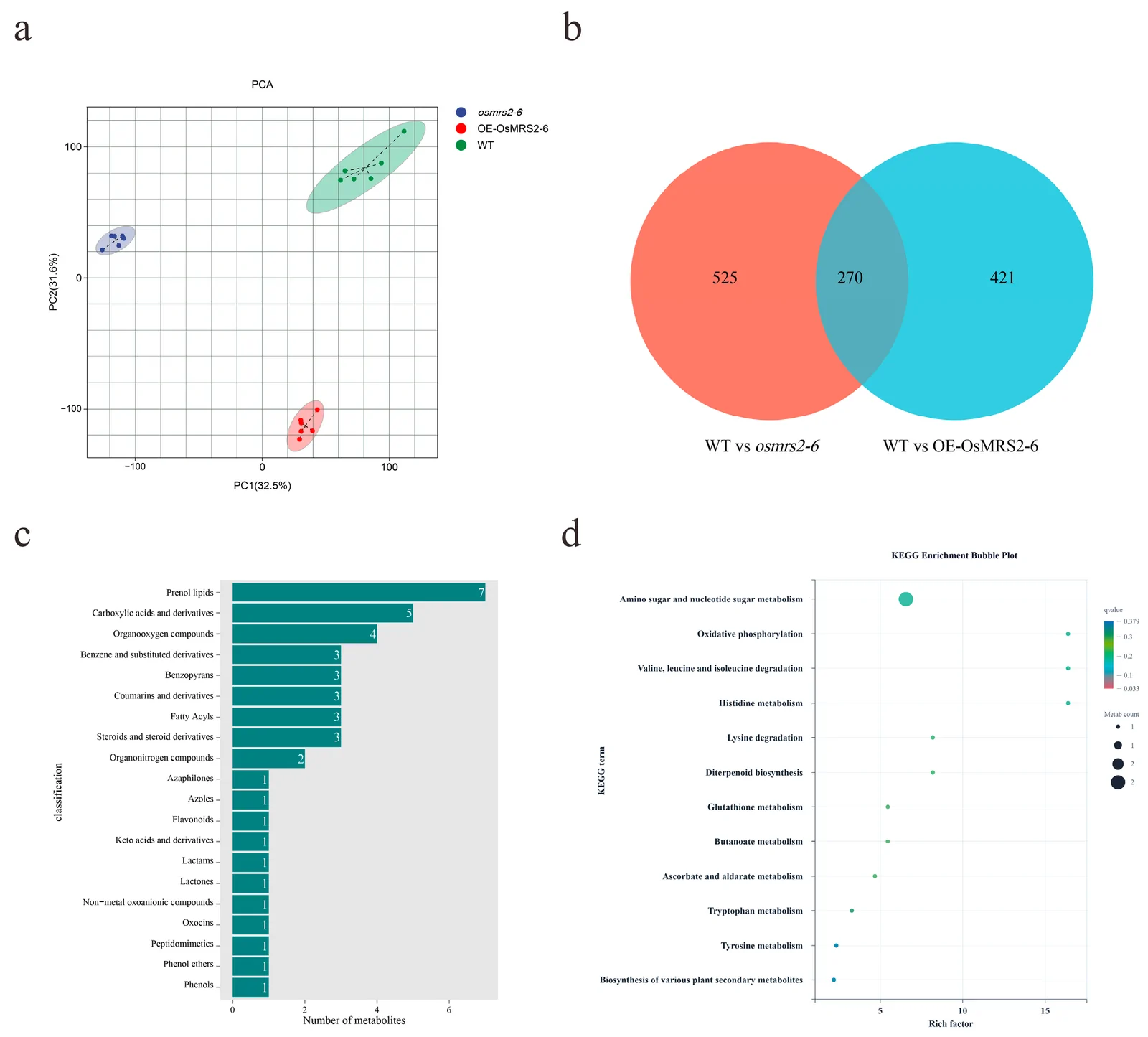
**Untargeted metabolomic analysis of leaves from WT, *osmrs2-6* and OE-OsMRS2-6 plants.** (**a**) Principal-component analysis (PCA) based on relative metabolite abundances. (**b**) Venn diagram showing the differential metabolites identified in the *osmrs2-6* versus WT and OE-OsMRS2-6 versus WT comparisons. (**c**) Chemical-class distribution of the 270 shared differential metabolites. (**d**) KEGG pathway-enrichment analysis of the shared differential metabolites.

**Figure 7:**
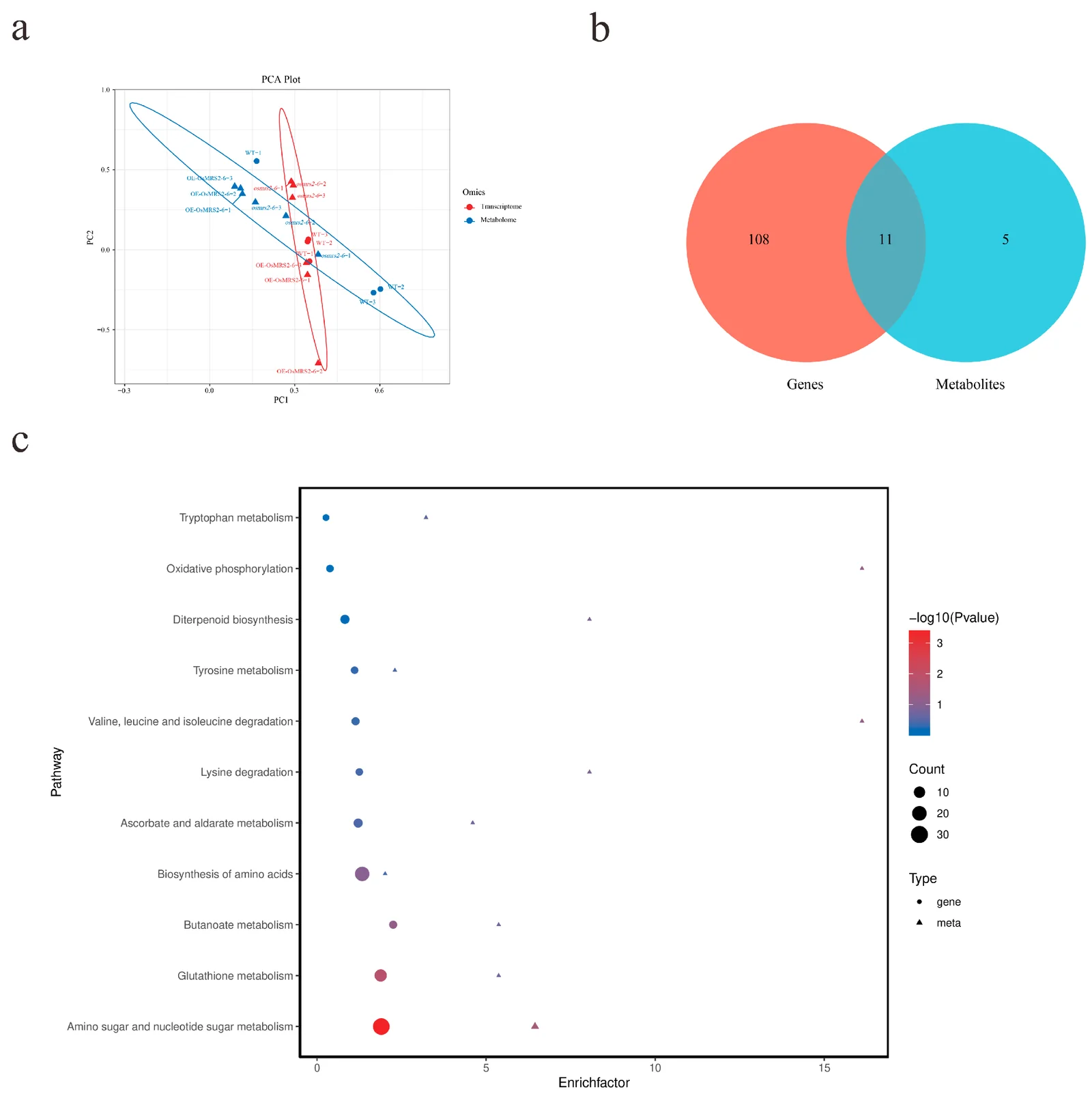
**Integrated transcriptomic and metabolomic analysis associated with *OsMRS2-6*.** (**a**) Joint principal-component analysis (PCA) of the transcriptomic and metabolomic datasets. (**b**) Venn diagram of the KEGG pathways identified from the transcriptomic and metabolomic datasets. (**c**) Integrated enrichment analysis of differentially expressed genes and differential metabolites across the 11 shared KEGG pathways.

**Table 1:** **Plastoglobule abundance in chloroplasts of different rice lines.**

Replicate	Plastoglobule Number (per Chloroplast)					
	WT	Mean	*osmrs2-6*	Mean	OE-OsMRS2-6	Mean
1	2, 3, 1, 5, 3, 0, 3	2.43	14, 2, 9, 3, 7, 5, 8	6.8	4, 2, 3, 3, 3, 4, 5	3.43
2	3, 8, 3, 4, 3, 1, 3	3.57	2, 7, 14, 6, 4, 14, 2	7	4, 2, 1, 0, 3, 1, 6	2.43
3	3, 3, 2, 3, 3, 3	2.83	13, 16, 2, 4, 3, 7	7.5	4, 6, 7, 7, 7, 0	5.17

**Table 2:** **Altered chloroplast RNA-editing efficiency in osmrs2-6 mutants.**

Chloroplast RNA-Editing Site	Editing Efficiency (%)			
	WT	*osmrs2-6-1*	*osmrs2-6-2*	*osmrs2-6-3*
*ndhB*-196	75	96	100	96
*ndhD*-293	79	65	57	58
*ndhF*-21	70	82	100	86
*rpoB*-182	51	100	99	100
*rpoB*-156	83	100	100	100
*rps8*-61	64	96	95	100
*rps3*-170	52	76	76	82
*rps3*-955	68	90	80	95

**Table 3:** **Number of bleached panicle tissues per plant in WT and OE-OsMRS2-6 overexpression lines.**

Replicate	Number of Bleached Panicle Tissues per Plant					
	WT-1	WT-2	WT-3	OE-OsMRS2-6-1	OE-OsMRS2-6-2	OE-OsMRS2-6-3
1	0	2	3	6	6	3
2	1	0	0	8	5	5
3	0	0	2	4	7	3

## Data Availability

Data will be made available on request.

## Supplementary Materials

The following supporting information can be downloaded at https://www.mdpi.com/article/10.3390/genes17101191/s1. Supplementary Table S1. Primer sequences used in this study; Supplementary Table S2. Protein accession numbers used for the phylogenetic analysis; Supplementary Table S3. Protein accession numbers for the rice OsMRS2 family used in the multiple-sequence alignment; Supplementary Table S4. Primers used for qRT-PCR analysis of chloroplast genes.
