# Supplementary material for: Integrated Transcriptomic and Metabolomic Analysis of OsMRS2-6 in Rice Growth and Chloroplast Traits
Source: Genes (Basel). 2026 Sep 26;17(10):1191. doi: 10.3390/genes17101191 (PMC13704160; doi:10.3390/genes17101191)
Supplement: Supplementary file 1 [file genes-17-01191-s001.zip › genes-4557350-supplementary.pdf]

Supplementary Table S1. Primer sequences used in this study

Name	Primer sequence (F)	Primer sequence (R)	Purpose
CR-MRS2-6-1	ggcATATTTCTTGGATTTAACCG	aaacCGGTTAAATCCAAGAAATA	OsMRS2-6 knockout
CR-MRS2-6-2	gccGCGGCCTGCGACGAGTACGA	aaacTCGTACTCGTCGCAGGCCG	OsMRS2-6 knockout
MRS2-6-1	TCGGAGGGCTCAACTTGCTC	AAGGCTGCACTGGTGGTTCA	Genotyping of knockout lines
MRS2-6-2	TGCCGTGTTAGGACGATGCG	CGCACTACGGGTGCTCAACA	Genotyping of knockout lines
PANMRS2-6	taagtccggagctagctctagaATGGCGTCCGTTTCGTC	cgcccttgctcaccatggatccggatccCAATATCTTTCTAGTC	Subcellular localization
OE-MRS2-6	tgcaggtcgactctagaggatccATGGCGTCCGTTTCGTC	ctcgcccttgctcaccatGGATCCCAATATCTTTCTAGTC	Overexpression construct
QRT-ACTIN	TGGCATCTCTCAGCACATTCC	TGCACAATGGATGGGTCAGA	Gene expression analysis
QRT-MRS2-6	gggttcacgagcaggtgatc	cgagcgtgcaatttcttccc	Gene expression analysis

Supplementary Table S2. Protein accession numbers used for the phylogenetic analysis

Species	Protein accession
Oryza sativa Japonica Group	NP_001405411.1
Zea mays	NP_001281185.1
Triticum aestivum	XP_044367954.1
Solanum lycopersicum	XP_004251000.1
Arabidopsis thaliana	NP_001332159.1
Physcomitrium patens	XP_024376179.1
Danio rerio	XP_073781978.1
Dictyostelium discoideum AX4	XP_643405.1

Supplementary Table S3. Protein accession numbers for the rice OsMRS2 family used in the multiple-sequence alignment

OsMRS2 family member	Protein accession
OsMRS2-1	NP_001389834.1
OsMRS2-2	NP_001383789.1
OsMRS2-3	NP_001392380.1
OsMRS2-4	NP_001410026.1
OsMRS2-5	NP_001404119.1
OsMRS2-6	NP_001405411.1
OsMRS2-7	NP_001405641.1
OsMRS2-8	XP_015635013.2
OsMRS2-9	NP_001406569.1

Supplementary Table S4. Primers used for qRT-PCR analysis of chloroplast genes

Gene name	Primer sequence (F)	Primer sequence (R)
atpA	TCGCGGAGTTACAAGCCTTT	ATTCTCGTAATCGTCGCCCG
atpB	GGGGAGCTGGAGTAGGTAA	CCCCTACTCCGCCAAATACG
atpE	ACTGACTCCTAAGCGAATT	GGCGTGGTTTTGGTAATAC
atpF-ex	AATTGCGTAGAGGAACCATT	TCTCGAGTATCCATTCATTGG
atpF-in	CGATCTCGACGAATTACTTC	GTGTTAATGGGTCTCATATTATTGG
atpH	CGTAGGCTCTTGCTTCTAT	GTCTCGCAATACCTTCTA
atpI	ATTAGTCGTTGTTGTTCTTGT	GCTTGAATACCGGCTTGTAA
cemA	TTTGCCTTGGGGAGTCTCTT	TTGTATTATTTCCCTTGCGTA
clpP	ATGTCAGCAGACGAAGCCAA	TCAGTATCGCAGTGCTCGTC
matK	TGCAATGGTTTGGGGTTCCGGTA	TGTGCGATCCACGAAAGAA
ndhA-ex	GATGCGGATATTTCTCTTTCT	TCTATGGACTGATATGGATTCT
ndhA-in	ACGACGGAAAGAAATAAAATAAGA	AATGGCTATCAAGAACGAATT
ndhB-ex	AAGTCCTACAGTGATGGATAAAG	CGGTCTAATGAGGCTACTATG
ndhB	TACGAAGGATCCCCACTCC	TCATCCTTCAGAGACTACGA
ndhB-in	AATCCTTCATCAGTGGTTGT	TCATCCTTCAGAGACTACGA
ndhC	CCCTTGGGCAATGAGTTTCG	CATTCCAAGGCTCCTTTTCGC
ndhE	TGGATTGATCACAAGCCGAAA	AGCAGCCGCAAGGGGCTATAA
ndhF	CTGCGGGGATTTTTCTTATA	GGCTAAGCTTCTTTTAATAT
ndhG	TCGCTAGGATTAGTTCTTGTT	TTAGACCATTCTGAGCCATT
ndhH	GTCCGACGCTTTAAAGAAA	AAGACTATCATCTCTACCA
ndhI	AATACGCGCAGCAAGGTACA	CCGGAAACGCTCCGATGTAA
ndhJ	TCCGATTTTCAAGAACGCGAAT	GGGCCAGCCTATCCAACTTT
ndhK	TCCTAGGCAAGCGGACCTAA	CAAGCTCCCATAGCAATGACG
petA	GCAGTGCTTCCCGATACTGT	AGCACCCACATTCAAACCCTC
petB	ACTGGAAGTGCTAGTGTGG	CGGAGTAAGCAGAGAAGGT
petD	CCGACGGGCTTATTGACAGT	GGTTGTAGCTACTGGGGCGAC
petL	CTAGTTATTTCGGTTTCTATTGG	GACGTATCTTGTTCCAAGCC
petN	GGATATAGTAAGTCTCGCTTGG	CTAGAGTCCACCTCCCTCC
psaA	GCAGTCGGATGTTTGGGGTA	GAAATCTCGGAGCACCCCAT
psaB	ATGAACGGACACCTTTGGCT	CCCACCAATCTTGCTTGCAC
psbC	GCTCCTTTAGGCTCTTTA	GTCGCTAACCAACTTCTA
psbD	GGCCCAATCGCTGTTTTTGT	CCAAAACTCGGCGCAAAGAA
psbE	CCCTATTCATTGCGGGTTGG	ACGGAATTCCTTGTGCGGTT
psbF	TTTTACAGTGCGATGGCTGG	CGTTGGATGAACTGCATTGCT
psbJ	TGGCCGATACTACTGGAAGA	CCCAATCCAGAATATGAACCGT
psbK	TTTCCTTCGCCCAAATTGCCCG	CAGCTTGCCAAACAAAAGGCT
psbL	ATGACACAATCAAACCCGAATG	ACAAAAATGAGTAATAAACCCAGT
psbM	ATATTCTCGCATTTATTGCTACTG	AATCATTTTTGGCTGACTGTTTT
psbN	GGAAACAGCAACTTTAGTCG	CGTGTTCTTCGAATGGATGCT

psbZ	GCTTCTCCTGATGGTTGGTTCA	AGCTACCAGAAAGACCAGTCC
rbcL	AGGGGAACGCGGAAATGACTT	AAAGATACCGCGAGCACGAT
rpl2-ex	ACGATTCTACCAGATATGTCTT	GGAACCATAGATAGGCAAGT
rpl2-in	CGTTCCGTCATCTGGCTTAT	TGATGGTCAGAGGCGGAATTG
rpl14	CAGTGCCCCAAATGCCTCTA	GCTGCATTGTGCGTCATAGCG
rpl16	ACGTATGGGTTCGGGAAAGG	CGGATACTCCGCCCATTCA
rpl22	TGGATGAGATTCGTTGGCGT	GAGTAGCATTTGCTGCTGCC
rpl23	AGCATTGGGGTCGAACTCTTCT	AGGGCCCATTCTTCTACCCCT
rpl36	TACAAAATGTGCTGATTGCGTAGG	ACCCCTGTCTTTTGTTATGCTTC
rpoA	CCGCCTTCCGTAGAAATCGT	CGCGATCTCTCTTGATCCGT
rpoB	GGCAGAAGAACTTTGAGAA	GGAAGAATAGGTGTGATTGATT
rpoC1	CGTGCTACTTCTAATGTT	CCTGTATGCCTAATCTATG
rpoC2	GGCTGATGATGAGGTAAG	TGAAGAATAGAAGGATAGACAA
rps8	CGACCTGGCTTACGAATTTA	ACTTCTCCCCCAAATTCTGTT
rps11	GCTGGTAGTGGGAAGAGAT	CGGCATAGGTGTTTACATC
rps12-ex	TTGCCAGAGTACGATTAACCT	TCACTCAAACTCCAAAGGTTAAACT
rps12-in	ATGTGAAGTGTATGGCTAACC	GCCCTTGTTGACGATTCTTT
rps14	GAGAGGAAGCGGCAGAAAT	CACGGACAAAGTAAGGGGAAA
ycf4	ATTATATCTGTACTTCCATCTCA	GTCATAACCACTGCCTAC
